# ST-segment elevation due to myocardial invasion of lung cancer mimicking ST elevation myocardial infarction: A case report

**DOI:** 10.1097/MD.0000000000026088

**Published:** 2021-05-21

**Authors:** Hae Won Jung

**Affiliations:** Department of Cardiology, Daegu Catholic University Medical Center, Daegu, Republic of Korea.

**Keywords:** lung cancer, metastasis, myocardium, ST-segment elevation

## Abstract

**Introduction:**

When a cancer patient presents with ST-segment elevation on an electrocardiogram (ECG), several causes including acute myocardial infarction (MI) should be considered. Myocardial metastasis is one of the rare causes of ST-segment elevation in cancer patients and its clinical silence makes it difficult to diagnose.

**Patient concerns:**

A 78-year-old man with lung cancer presented to the emergency room for chest pain. ECG revealed ST-segment elevation in inferior and lateral leads.

**Interventions:**

After emergent coronary angiography, percutaneous coronary intervention (PCI) on proximal right coronary artery was performed.

**Outcomes:**

Even 7 days after PCI, ST-segment elevation in inferior and lateral leads still existed. Cardiac markers continued to be within the normal range.

**Diagnosis:**

We found evidence of metastasis of lung cancer on the inferolateral wall of the myocardium by trans thoracic echocardiogram and positron emission tomography (PET)/computed tomography (CT). We diagnosed myocardial metastasis as the cause of ST-segment elevation in the patient.

**Conclusion:**

Myocardial metastasis is one of the differential diagnosis of ST-segment elevation in cancer patients. Periodic ECG is necessary for lung cancer patients and rapid cardiac work-up is recommended when ST-segment elevation is newly discovered.

## 1 Introduction

Most cancers were associated with an increased risk of coronary heart disease.^[1]^ Therefore, it is important to carefully observe the electrocardiogram (ECG) change in cancer patients. When a cancer patient presents with ST-segment elevation on ECG, several causes including myocardial infarction (MI) should be considered. Metastatic involvement of the heart is one of the differential diagnosis of ST-segment elevation in cancer patients. It features persistent ST-segment elevation without typical ECG changes of infarction.^[2]^ Here, we present a rare case of 1 patient with ST-segment elevation due to myocardial invasion of lung cancer mimicking ST-segment elevation myocardial infarction (STEMI).

## 2 Case presentation

**Figure 1:**
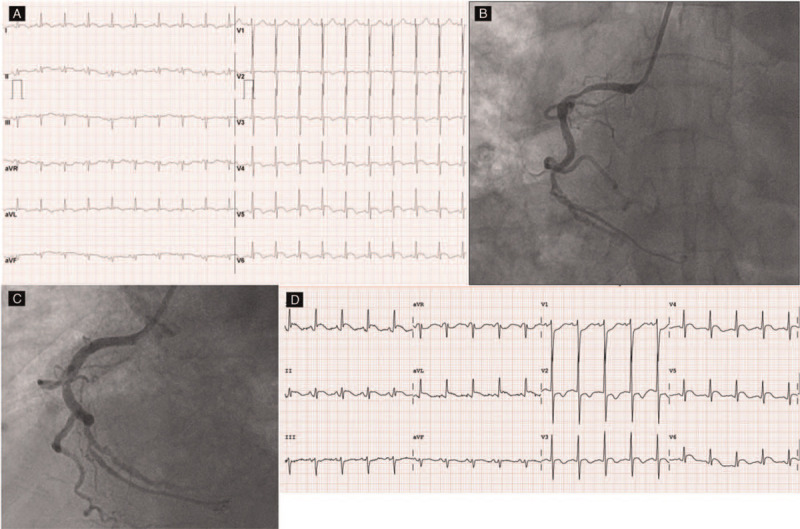
ECG at ER with ST-segment elevation at inferior and lateral leads (A). Emergent CAG showed significant stenosis on proximal RCA (B). CAG after PCI on proximal RCA (C). ECG 7 days after PCI: ST-segment elevation persisted at inferior and lateral leads (D). CAG = coronary angiography, ECG = electrocardiogram, ER = emergency room, PCI = percutaneous coronary intervention, RCA = right coronary artery.

**Figure 2:**
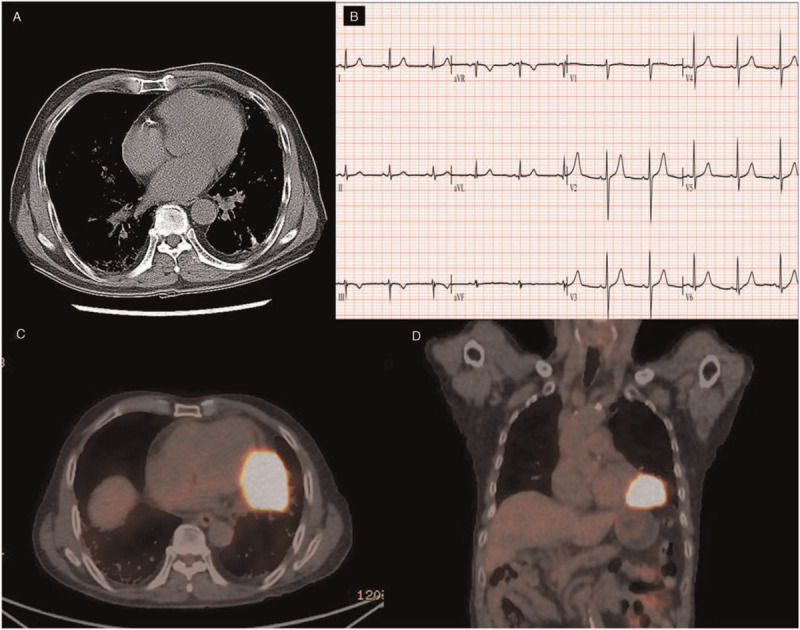
Chest CT 1 year ago, there was no visible lung mass (A). ECG 1 year ago, there was no ST-segment elevation (B). PET CT 6 month ago, the myocardium was invaded by intense hypermetabolic mass in the left lower lobe (C, D). CT = computed tomography, ECG = electrocardiogram, PET = positron emission tomography.

**Figure 3:**
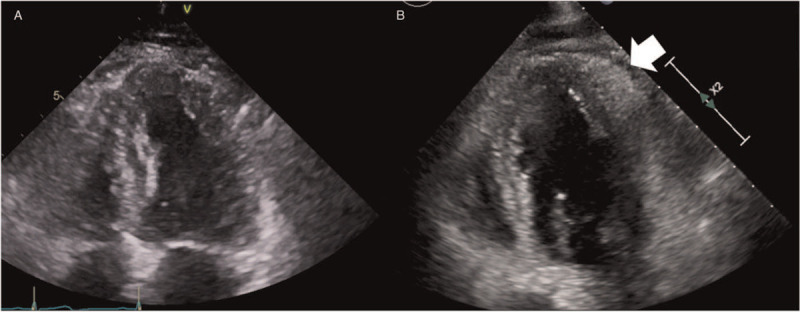
TTE 1 year ago was a normal study (A). TTE after PCI showed focal areas of myocardial thickening and associated hypokinesis with an adherent, mobile echo density attached to the inferolateral myocardium (B). TTE = trans thoracic echocardiogram, PCI = percutaneous coronary intervention.

A 78-year-old male patient visited emergency room (ER) with ongoing chest pain and dyspnea. Blood pressure was 140/80 mmHg and heart rate were 120 beats per minute. ECG showed ST-segment elevation in inferior and lateral leads (Fig. 1A). He was diagnosed with stage T4 squamous cell lung cancer 6 months ago and was undergoing radiation therapy. He also had idiopathic pulmonary fibrosis. We performed emergent coronary angiography and found significant stenosis on proximal right coronary artery (Fig. 1B). We deployed drug-eluting stent (3.25 × 15 mm) on proximal right coronary artery (Fig. 1C). The procedure was successful. The stent in the right coronary artery was fully expanded and there was no residual stenosis. After percutaneous coronary intervention (PCI), his chest pain was relieved; however, ST-segment elevation in inferior and lateral leads still existed even 7 days after PCI (Fig. 1D). There was no cardiac marker elevation before and after PCI. We reviewed his medical records and confirmed that his ECG had no ST-segment elevation 1 year ago when lung mass was not visible on the chest CT (Fig. 2A and B). In positron emission tomography/computed tomography (PET/CT) 6 months ago, the myocardium was invaded by intense hypermetabolic mass in the left lower lobe (Stage T4) (Fig. 2C and D). Trans thoracic echocardiogram (TTE) after PCI also showed focal areas of myocardial thickening and associated hypokinesis with an adherent, mobile echo density attached to the inferolateral myocardium which was not seen in TTE 1 year ago. There was no pericardial effusion on TTE after PCI (Fig. 3A and B). Taking the above test results together, we concluded the cause of the patient's ST-segment elevation is myocardial involvement of lung cancer. Chest pain was relieved after PCI, however, pneumonia developed days after PCI. Medical treatment including antibiotics was started. Unfortunately, the patient died a month after PCI due to respiratory failure with pneumonia. The ST-segment elevation on inferior and lateral leads was persistent until the patient died.

## 3 Discussion and conclusion

Cardiac metastases are considered to be rare. However, the incidence of cardiac metastases reported in the literature varies widely from 2.3% to 18.3%.^[3]^ The main causes for cardiac metastasis are melanoma, carcinoma (lung, breast, esophagus, rarely colon, and rectum), and hematologic malignancies (leukemia and lymphoma). Possible pathways for cardiac tumor spread are hematogenous pathway, lymphatic pathway, transvenous pathway, and direct invasion.^[4]^ The pericardium is the most frequently involved site of cardiac metastasis, comprising 64% to 69% of all cardiac metastases. Epicardial involvement and myocardial involvement represent the second and third most common sites of cardiac metastasis.^[5]^ Most cardiac metastases are clinically silent and are often diagnosed only after death. Therefore, early detection of cardiac metastases is challenging.^[4]^

ECG can be a useful to diagnose cardiac metastases. Although it is a non-specific test, it has the advantage of being non-invasive and can be performed frequently. More than two-thirds of patients with cardiac metastasis had ECG abnormalities.^[6]^ Cates et al^[7]^ reported that patients with cardiac metastases had a significantly higher frequency of atrial arrhythmia, low voltage, and myocardial ischemia on ECG compared with patients without cardiac metastases, and that cancer patients with normal ECG were less likely to have cardiac metastases.

ECG finding of localized and prolonged ST-segment elevation without Q waves have a high specificity for myocardial tumor invasion.^[6]^ Several case reports of cardiac metastasis mimicking STEMI have been reported.^[2,8–10]^ For these reasons, periodic ECG is necessary for cancer patients and rapid cardiac work-up is recommended especially, when ST-segment elevation is newly discovered. If ST-segment elevation is newly found in cancer patients, STEMI, takotsubo cardiomyopathy, myopericarditis, myocardial metastasis, hyperkalemia, pulmonary embolism should be considered as differential diagnoses.^[2]^ Still, the mechanism of ST-segment elevation in myocardial metastasis have not been fully understood. However, it characterized by persistent ST-segment elevation without typical ECG changes of MI such as the development of Q waves in consecutive ECGs^[2]^ and the leads with ST-segment elevation may reflect the location of the myocardial involvement, similar to those of STEMI patients.^[9]^ Like our patient, in most cases of previously reported ST-segment elevated cardiac metastases, cardiac enzymes were normal.^[9]^ Normal cardiac enzyme levels can be a distinguishing feature in patients with ST-segment elevation due to myocardial metastasis compared with patients with STEMI.

Evaluating cardiac metastasis, often requires a multimodality imaging approach including echocardiography, cardiac magnetic resonance imaging (MRI), cardiac CT, fluorodeoxyglucose-18-(FDG) PET/CT. Echocardiography is the initial imaging modality to detect pericardial effusions and to assess the presence of any cardiac metastasis and the hemodynamics.^[5]^ Cardiac MRI is the best imaging modality for evaluating the extent of myocardial involvement by metastatic disease. The tissue characterization feature of cardiac MRI can be used to differentiate invasive metastases from the myocardium and blood clots from tumors.^[11]^ Cardiac CT has a lower contrast resolution than cardiac MRI, but offers superior spatial resolution. Like cardiac MRI, cardiac CT can identify direct tumor extension from adjacent mediastinal structures.^[11]^ The advantage of Fluorine-18-FDG PET/CT is that it can improve the detection of distant extra-cardiac metastatic disease by imaging the whole body and it can help to distinguish some malignant tumors from benign ones.^[5]^

Cardiac metastases have a poor prognosis. Even compared with primary malignant heart tumor, the prognosis of cardiac metastasis is worse. Hoffmeier et al^[12]^ reported patients with primary malignant heart tumor survived 5.5 years on average after radical surgical resection and patient with cardiac metastases survived 1.5 years on average after radical surgical resection. Cardiac metastases are most often found in patients with multiple metastases and a high burden of disseminated disease. Therefore, the most important goals of treatment should include the symptom relief and prevention or delay of symptom recurrence.^[5]^ Surgical excision is main treatment of primary or metastatic malignancies of the heart and great vessels for the symptom relief and hemodynamic improvement.^[13]^ However, Park et al^[14]^ suggested that aggressive surgical management of thoracic malignancies invading heart and great vessel can derive potential for cure. Oh et al^[13]^ reported 2 patients of cardiac metastases (colon cancer, lung cancer) who successfully treated with aggressive surgical excision and adjuvant chemotherapy. Since they had the cardiac metastases without residual cancer at the primary origin sites, the main purpose of surgery was cure. There was no evidence of disease status in these patients during the follow up period. Surgical treatment for cardiac metastases is very limited, however, it seems that a good prognosis can be expected if the patient is well screened and complete resection is performed. Radiotherapy and chemotherapy can also be useful treatments of certain cardiac metastases. Therefore, it is important to involve a multidisciplinary team in the management of the patient with cardiac metastasis.^[5]^

This case showed the relationship between myocardial invasion and ST-segment elevation in chronological order and showed that the ST-segment elevation due to myocardial metastasis persisted over time even after PCI. In addition, this case also showed that the leads with ST-segment elevation may reflect the location of the myocardial involvement, similar to those of STEMI patients

## 4 Consent

Informed written consent was obtained from the patient and his family for the publication of this case report and any accompanying medical images.

## Acknowledgments

The author thanks the patient and his family for permission to publish this case report.

## Author contributions

**Conceptualization:** Hae Won Jung.

**Data curation:** Hae Won Jung.

**Resources:** Hae Won Jung.

**Software:** Hae Won Jung.

**Supervision:** Hae Won Jung.

**Validation:** Hae Won Jung.

**Visualization:** Hae Won Jung.

**Writing – original draft:** Hae Won Jung.

**Writing – review & editing:** Hae Won Jung.
